# Metagenomic Snapshots of Viral Components in Guinean Bats

**DOI:** 10.3390/microorganisms9030599

**Published:** 2021-03-15

**Authors:** Roberto J. Hermida Lorenzo, Dániel Cadar, Fara Raymond Koundouno, Javier Juste, Alexandra Bialonski, Heike Baum, Juan Luis García-Mudarra, Henry Hakamaki, András Bencsik, Emily V. Nelson, Miles W. Carroll, N’Faly Magassouba, Stephan Günther, Jonas Schmidt-Chanasit, César Muñoz Fontela, Beatriz Escudero-Pérez

**Affiliations:** 1 Morcegos de Galicia, Magdalena G-2, 2o izq, 15320 As Pontes de García Rodríguez (A Coruña), Spain; robertox.hermida@gmail.com; 2 WHO Collaborating Centre for Arbovirus and Haemorrhagic Fever Reference and Research, Bernhard Nocht Institute for Tropical Medicine, 20359 Hamburg, Germany; danielcadar@gmail.com (D.C.); bialonski@bnitm.de (A.B.); baum@bnitm.de (H.B.); hhakamak@emich.edu (H.H.); bencsik@bnitm.de (A.B.); nelson@bnitm.de (E.V.N.); guenther@bni.uni-hamburg.de (S.G.); jonassi@gmx.de (J.S.-C.); munoz-fontela@bnitm.de (C.M.F.); 3 Laboratoire des Fièvres Hémorragiques en Guinée, Université Gamal Abdel Nasser de Conakry, Commune de Matoto, Conakry, Guinea; koundounofr@yahoo.fr (F.R.K.); cmagassouba01@gmail.com (N.M.); 4 Estación Biológica de Doñana, CSIC, 41092 Seville, Spain; juste@ebd.csic.es (J.J.); juanele@ebd.csic.es (J.L.G.-M.); 5 CIBER Epidemiology and Public Health (CIBERESP), 28029 Madrid, Spain; 6 Public Health England, Porton Down, Wiltshire SP4 0JG, UK; Miles.Carroll@phe.gov.uk; 7 Wellcome Centre for Human Genetics, Nuffield Department of Medicine, Oxford University, Oxford OX3 7BN, UK; 8 German Centre for Infection Research (DZIF), Partner Site Hamburg-Luebeck-Borstel, 38124 Braunschweig, Germany; 9 Faculty of Mathematics, Informatics and Natural Sciences, Universität Hamburg, 20148 Hamburg, Germany

**Keywords:** bats, host, zoonosis, Ebola virus, nonretroviral integrated RNA viruses (NIRVs)

## Abstract

To prevent the emergence of zoonotic infectious diseases and reduce their epidemic potential, we need to understand their origins in nature. Bats in the order Chiroptera are widely distributed worldwide and are natural reservoirs of prominent zoonotic viruses, including Nipah virus, Marburg virus, and possibly SARS-CoV-2. In this study, we applied unbiased metagenomic and metatranscriptomic approaches to decipher the virosphere of frugivorous and insectivorous bat species captured in Guéckédou, Guinea, the epicenter of the West African Ebola virus disease epidemic in 2013–2016. Our study provides a snapshot of the viral diversity present in these bat species, with several novel viruses reported for the first time in bats, as well as some bat viruses closely related to known human or animal pathogens. In addition, analysis of *Mops condylurus* genomic DNA samples revealed the presence of an Ebola virus nucleoprotein (NP)-derived pseudogene inserted in its genome. These findings provide insight into the evolutionary traits of several virus families in bats and add evidence that nonretroviral integrated RNA viruses (NIRVs) derived from filoviruses may be common in bat genomes.

## 1. Introduction

Due to their biodiversity, rainforest areas of Central and Western Africa are considered hotspots for the emergence of zoonotic viruses, and a number of prominent viruses with epidemic potential have been identified in this region [1]. Approximately 75% of emerging infectious diseases in humans are zoonoses [2,3]. The rate of detection of zoonotic viruses has increased in past decades, possibly due to improved diagnostic capacity and surveillance efforts [4,5]. Many novel pathogens that have caused epidemics and pandemics have emerged from bats, including Nipah virus, MERS-coronavirus, Marburg virus, and likely Ebola virus (EBOV) and SARS-CoV-2 [6].

Due to the importance of bats as virus reservoirs, it is paramount to regularly investigate the bat virome and to assess the potential human pathogenicity of viruses circulating in bats. In this regard, metagenomic analyses of bat viromes can provide relevant information about viruses circulating in frugivorous and insectivorous bats living in a specific area. Subsequent phylogenetic analyses can evaluate the proximity of bat viruses to known pathogenic human viruses, which may help gauge potential spillover events into humans. For instance, potential novel variants of paramyxovirus and coronaviruses have been shown to commonly circulate in bats [7,8].

In addition, metagenomic analyses of bat viromes have served, for example, to identify novel filoviruses such as Bombali virus in *Mops condylurus* [9] and Mengla dianlovirus in *Rousettus* bats [10,11,12,13], which provides evidence for bats as reservoirs for Ebola virus (EBOV). Furthermore, EBOV RNA has been detected in three fruit bat species: *Epomops franqueti, Hypsignathus monstrosus*, and *Myonycteris torquata* [14]. Anti-EBOV antibodies have been shown in those species, as well as in *Eidolon helvum, Epomophorus gambianus, Micropteropus pusillus, Mops condylurus, Rousettus aegyptiacus*, and *Rousettus leschenaultii* [15].

Finally, in silico analyses of mammalian genomes in the order Mononegavirales have identified nonretroviral sequences derived from single-strand RNA viruses (NIRVs) that are integrated into the genomes of several mammalian species, including bats. Of these, bornavirus NIRVs are the best-characterized [16,17]. Filovirus-derived pseudogenes have also been identified in the genome of bats, marsupials, and rodents [18]. These NIRVs are thought to have their origin in nonhomologous recombination events with genomic transposons during infection [19,20,21]. Because phylogenetic studies show that these sequences are essentially paleovirus sequences, these findings indicate that filoviruses are ancient and have a long relationship with these mammalian species.

In this study, bats captured in the rainforest area of Guéckédou in the Republic of Guinea were sampled via metagenomic and metatranscriptomic studies to characterize the virome of these bat species. In addition, genomic DNA samples were also screened for the presence of possible filovirus-derived nonretroviral integrated RNA virus (NIRV) sequences. The overall goal was to gain insight into the viruses circulating in bats in the area where the 2013–2016 Ebola virus disease (EVD) epidemic started and to underscore the importance of bat surveillance to prevent potential zoonotic outbreaks.

## 2. Materials and Methods

### 2.1. Bat Capturing and Sampling

**Figure 1:**
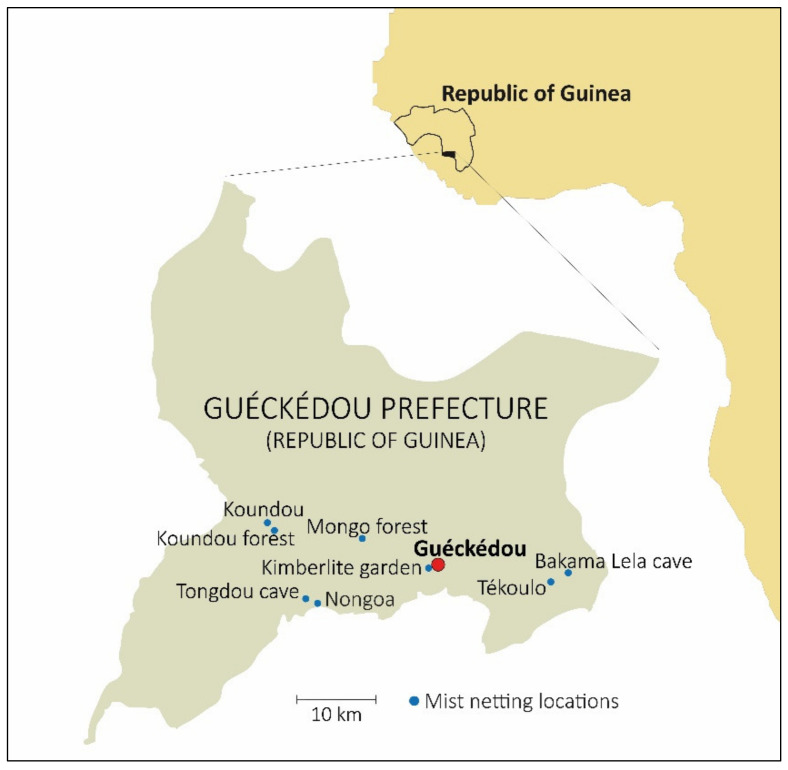
Locations of the bat sampling sites. Bats were sampled in eight locations in the Guéckédou prefecture: Guéckédou (Kimberlite garden), Tékoulo (Tékoulo village and Bakama Lela cave), Temessadou (Mongo forest), Nongoa (Nongoa village and Tongdou cave), and Koundou-Lengobengou (Koundou village and Koundou forest).

The objectives of the present study were communicated to the local community leaders in the Guéckédou prefecture, as well as to the regional government. This study was approved the 7 February 2017 by the Ministère de l’Elèvage et des Productions Animals and the Direction Nationale des Services Vétérinaires de la Republique de Guinée under permit number 015/MEPA/DNSV/2016. The capture and handling of animals and samples were conducted only by trained individuals. Personal safety equipment for capture included leather gloves, goggles, and masks. Bats were sampled in eight locations in the Guéckédou prefecture over the course of five days, between 10 and 15 April 2017. Bats were captured with 9 m and 12 m mist nets at different heights in Guéckédou (Kimberlite garden), Tékoulo (Tékoulo village and Bakama Lela cave), Temessadou (Mongo forest), Nongoa (Nongoa village and Tongdou cave), and Koundou-Lengobengou (Koundou village and Koundou forest) (Figure 1). Different environments were sampled: two caves, two construction sites, two secondary forest zones, and two rural core environments. A total of 82 bats were captured by mist nets and kept in cloth bags until sample collection. The forearm length and weight of each specimen were measured. Bats were released after the authors took a blood sample (for virus detection) and a patagium sample (for species identification); these were conserved in AVL and 70% ethanol, respectively. One specimen with a broken wing was euthanized under isoflurane anesthesia and cervical dislocation. Thirteen bats were found dead due to stress. For these 14 specimens, necropsies were performed to obtain spleen, liver, kidney, and thymus samples, which were preserved in 500 μL of RNA*later*. Samples were stored initially at −20 °C and later at −80 °C. Necropsies were carried out wearing an all-over bodysuit (Tyvek), FFP3 safety mask, face shield, arm protection, and doubled gloves. All other nondisposable equipment was disinfected with 90% ethanol. Nets were dried and disinfected every morning. Bat carcasses were burned after sampling.

### 2.2. Molecular Identification of the Bat Species

Bats were initially identified in the field based on morphology, using an ad hoc guide that compiled the available information for bats in the area. Molecular identification was done at the Molecular Ecology Laboratory of the Doñana Biological Station (Sevilla, Spain). For the molecular identification of the species, total DNA extraction was performed from the patagium sample conserved in 70% ethanol. Total DNA was isolated and purified by saline precipitation of proteins and capture of DNA with isopropanol. Once total DNA was extracted and purified, two mitochondrial fragments of the extracted DNA were amplified by PCR. A fragment of approximately 380 bp of the *cytochrome oxidase subunit 1* (COI) gene (*MT-COI*) was amplified with mlCOIintF/mlCOIintR primers [22]. Thermocycling consisted of 16 initial cycles with denaturation for 10 s at 95 °C, annealing for 30 s at 62 °C (−1 °C per cycle), and extension for 60 s at 72 °C, followed by 25 cycles at 46 °C annealing temperature. Another fragment of approximately 650 bp of the *cytochrome b* (Cytb) gene (*MT-CYB*) was amplified with the primers MolcitF [23] /MVZ16 [24]. Thermocycling comprised a 4 min initial denaturation at 94 °C, followed by 35 cycles of 45 s at 94 °C, 45 s at 50 °C, and 60 s at 72 °C. Finally, there was an extension of 10 min at 72 °C. PCR amplifications were checked on 1.5% agarose gels. Both fragments were sequenced by the Sanger method in the automatic sequencer ABI 3100 (Applied Biosystems). These sequences were compared to databases of previously published sequences via BLAST (https://blast.ncbi.nlm.nih.gov/Blast.cgi, accessed on 18 February 2021) and BOLD (Barcode of Life Data System; https://www.boldsystems.org/, accessed on 18 February 2021). Bat species identification was based on a 98% of similitude threshold for the larger Cytb fragment. The identification was based on the COI fragment only when the Cytb was not available for the species.

### 2.3. Sample Processing and Characterization of the Bat Virome

Viral RNA was extracted using QIAamp Viral RNA Mini Kit (QIAGEN, Hilden, Germany) according to the manufacturer’s protocol. Bat organ samples were individually cut into small pieces and digested for 30 min at 37 °C in DMEM with 10% containing collagenase D. Digested organs were mashed through a 70 μm cell strainer into PBS. After centrifugation, supernatant was discarded, and the cell pellet was resuspended in AVL buffer and processed for RNA extraction. The extracted viral RNA/DNA was subjected to next-generation sequencing (NGS). After random RT-PCR amplification of the RNA, the extracted viral DNA and RNA were subjected to library preparation using a QIAseq FX DNA Library Kit (QIAGEN, Hilden, Germany). Normalized samples were pooled and sequenced using 300-cycle (2 × 150 bp paired-end) NextSeq 550 reagent kits v2.5 (Illumina, San Diego, CA, USA) on a NextSeq 550 platform. The generated raw data were cleaned from low-quality reads and polyclonal sequences were removed. The curated sequence data were then compared with viral databases and nonredundant proteins (https://www.ncbi.nlm.nih.gov/refseq/about/nonredundantproteins, accessed on 18 February 2021; https://www.ncbi.nlm.nih.gov/genome/viruses/), accessed on 18 February 2021. The cut-off E-value for the BLASTx analyses and comparison was set to 0.001. The viral metagenomic and metatranscriptomic output was visualized and analyzed in MEGAN [25].

### 2.4. Sequence Data and Phylogenetic Analysis

Sequence analysis, genome assembly, genomic organization, and multiple alignments of the viruses detected were performed using Geneious Prime (Biomatters, Auckland, New Zealand). Open reading frames (ORFs) were predicted with Geneious and ORFfinder (https://www.ncbi.nlm.nih.gov/orffinder/, accessed on 18 February 2021). Nucleotide or amino acid sequences of partial genomes of the newly characterized virus species were aligned with the close relative reference viruses of the corresponding virus family or species. The nucleotide or amino acid sequences were aligned using the E-INS-i algorithm implemented in MAFFT (https://mafft.cbrc.jp/alignment/software/algorithms/algorithms.html, accessed on 18 February 2021). Evolutionary relationships of the detected viruses were analyzed by constructing phylogenetic trees using the maximum likelihood method in PhyML 3.0 [26], with subtree pruning and regrafting (SPR) branch-swapping and an approximate likelihood ratio test (aLRT) for assessment of specific node support.

### 2.5. Identification of Nonretroviral Integrated RNA Virus (NIRV) Elements

Genomic DNA from bat spleen samples was subjected to PCR using NIRV universal primers directed against conserved regions of NIRVs found in marsupials, rodents, and bats (NIRVfw: aggtggagcctgtcttgaaa and NIRVrv: atcacatcctgatggctggt). PCR was performed using the GoTaq G2 Hot Start Green Master Mix kit (Promega) under the following thermal cycling conditions: 95 °C for 2 min followed by 37 cycles at 95 °C for 30 s, 43 °C for 1 min and 72 °C for 30 s, and a final step of 72 °C for 5 min. Amplification products of the expected size of 276 bp were purified using QIAquick PCR Purification kit or QIAquick Gel Extraction kit (QIAGEN, Hilden, Germany) according to manufacturer’s instructions and then sequenced using the Sanger method. Identification of open-reading frames (ORFs) and translated products was done using Expasy (https://www.expasy.org/, accessed on 18 February 2021). Multiple protein sequence analysis was carried out via Clustal Omega at EMBL-EBI. Alignment visualization was done using MView (https://desmid.github.io/mview/, accessed on 18 February 2021).

## 3. Results

### 3.1. Bat Species Identification

Bats were sampled in eight locations (Figure 1) between 10 and 15 April 2017. A total of 82 specimens were captured and 11 species were genetically identified (Table 1).

### 3.2. Characterization of the Bat Virome

To search for virus sequences, we utilized bat necropsy sample pools consisting of RNA samples from spleen, liver, kidney and, in some cases, thymus from 14 individual bats belonging to five species (Table 2). Metatranscriptomic and metagenomic sequencing resulted in 9 to 29 million reads per pool (185,569,458 reads in total), which were assembled de novo into 17,730 to 49,403 contigs. BLAST analyses of sequence reads from the metatranscriptomic and metagenomic protocol revealed large proportions of reads from Bacteria (3.57% to 17.53%), with 48.32% to 72.3% from Eukaryota and only 0.3% to 1.4% from viruses.

**Figure 2:**
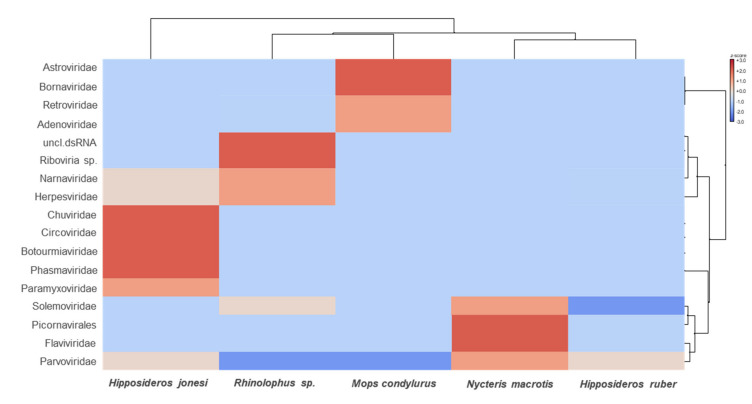
Heat map visualizing the most abundant virus families in each pooled bat sample. The bat species are listed in the bottom text row. The names of the virus families are presented in the left text column. Heat maps are color-coded based on row z-scores.

**Figure 3:**
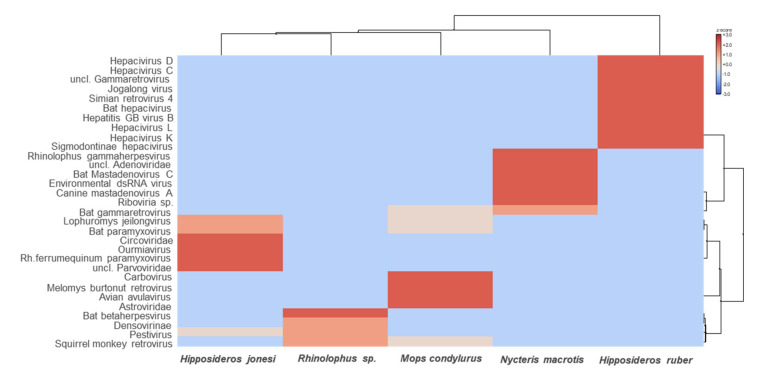
Heat map visualizing the most abundant virus species in each pooled bat sample. The bat species are listed in the bottom text row. The names of the virus species are presented in the left text column. Heat maps are color-coded based on row z-scores.

Overall, viruses from a wide range of viral families were detected, of which 84.13% to 91.36% were RNA viruses and 8.64% to 15.87% were DNA viruses. Metatranscriptomics revealed high levels of viral diversity, the most abundant viral groups detected being *Astroviridae, Bornaviridae, Retroviridae, Adenoviridae, Narnaviridae, Herpesviridae, Chuviridae, Circoviridae, Botourmiaviridae, Phasmaviridae, Paramyxoviridae, Solemoviridae, Picornaviridae, Flaviviridae*, and *Parvoviridae* (Figure 2 and Figure 3).

**Figure 4:**
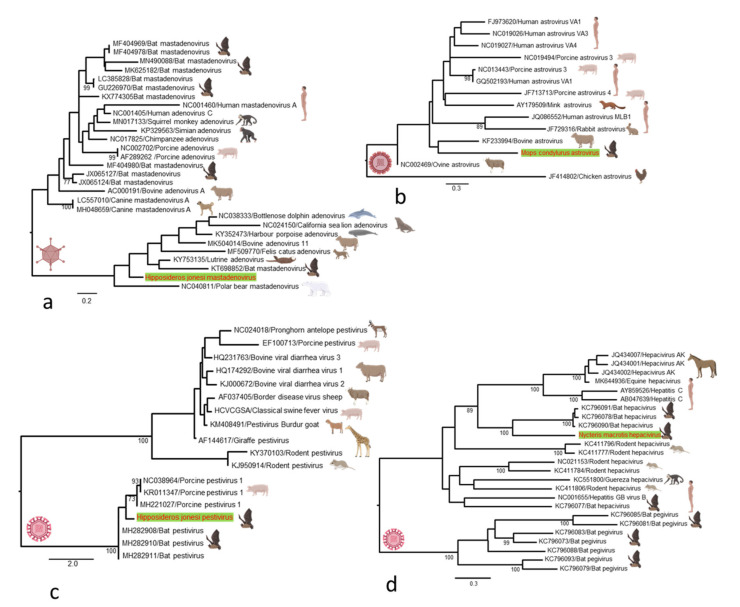
Maximum likelihood phylogenetic trees of viruses detected with representative members that were previously identified in other mammalian and avian hosts. (**a**) Phylogenetic analysis of representative adenovirus species based on the 353 nucleotide (nt) sequence of DNA polymerase gene. (**b**) Phylogenetic analysis of representative astroviruses based on the 267 bp sequence of the ORF1a gene. (**c**) Phylogenetic analysis based on complete P7 gene nucleotide sequences of pestivirus detected in this study compared with other previously identified representatives. (**d**) Phylogenetic analysis based on the 1453 bp sequence of the hepacivirus RdRp gene. All trees are midpoint rooted and scaled to nucleotide substitutions per site. Bootstrap values (70%) are shown at the key nodes. The viral sequences detected in this study are shown in red in each tree. The silhouettes of figures were created with BioRender.com.

*Adenoviruses.* Adenovirus (AdV)-related sequences were detected in one metagenomic library. We identified partial genome sequence with 76.4% pairwise nucleotide identity, tentatively named *Hipposideros jonesi* mastadenovirus. Phylogenetic analysis based on partial DNA polymerase gene sequences showed that *Hipposideros jonesi* mastadenovirus formed a distinct cluster that is more closely related to mastadenoviruses of bats from China and polar bears, and latrine mastadenoviruses than to those previously described in bats, which are evolutionary in separated groups (Figure 4a).

*Astroviruses*. *Mamastrovirus* in the family Astroviridae infects many mammals, including bats and humans, and causes gastroenteritis. In this study, astrovirus-related sequences were detected in *Mops condylurus* and showed a nucleotide identity similarity of 76.2% with a human astrovirus previously reported in Kenya. We constructed a phylogenetic tree based on partial ORF1a gene to show the evolutionary relationship between *Mops condylurus* astrovirus and other representative astroviruses. Phylogenetic analysis suggested that the *Mops condylurus* astrovirus forms a distinct cluster most closely related to human astrovirus (Figure 4b).

*Pestiviruses.* A fragmented genome of a novel species of pestivirus was identified in one *Hipposideros jonesi* metatranscriptomics library. The genomic fragments share 78% similarity, tentatively named *Hipposideros jonesi* pestivirus. Phylogenetic analysis based on complete P7 gene nucleotide sequences showed that the novel pestivirus forms a distinct cluster with porcine and bat pestiviruses previously reported in China (Figure 4c). While the bat pestiviruses are clustered together in the phylogenetic tree, their relationship with viruses identified from mammals other than swine remains unresolved.

*Hepaciviruses*. In one *Nycteris macrotis* metatranscriptomic library, we identified 32 contigs matching different regions of a novel hepacivirus genome, provisionally named *Nycteris macrotis* hepacivirus. In a phylogenetic reconstruction based on partial RdRp gene, it clustered with a distinct group of bat-associated hepaciviruses but formed a distinct lineage within the group, likely indicating a common ancestor (Figure 4d). A BLAST search of the RdRp gene showed that *Nycteris macrotis* hepacivirus exhibited the greatest nucleotide similarity (56.3%) with bat hepaciviruses isolated previously from large-eared free-tailed bats (*Otomops martiensseni*) from Kenya.

**Figure 5:**
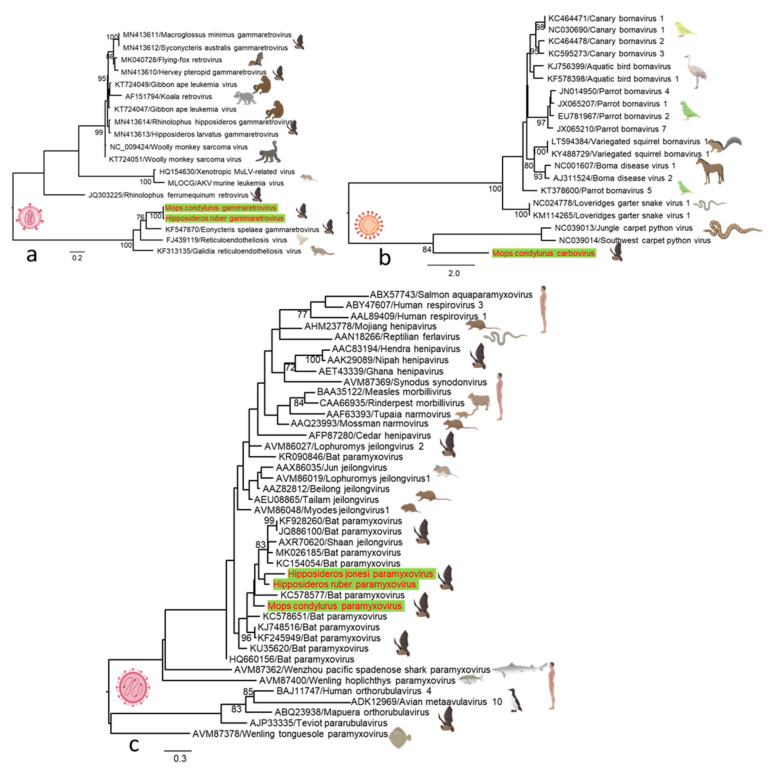
Maximum likelihood phylogenetic trees of viruses detected with representative members that were previously identified in other mammalian and avian hosts. (**a**) Phylogenetic analysis of representative gammaretroviruses based on the 408 nucleotide (nt) sequence of a group-specific antigen (gag) gene. (**b**) Phylogenetic analysis of representative *Bornaviridae* based on partial RdRp amino acid sequences. (**c**) Phylogenetic analysis based on partial RdRp amino acid sequences of the novel paramyxovirus detected in this study with other previously identified representative members of *Paramyxoviridae*. The phylogenetic trees are midpoint rooted and the bar scales represent either the nucleotide or amino acid substitutions per site. Bootstrap values (70%) are shown at the key nodes. The new virus species detected in this study is shown in red in each tree. The silhouettes of figures were created with BioRender.com.

*Gammaretroviruses*. Gammaretrovirus-related sequences were detected in the *Mops condylurus* and *Hipposideros ruber* metatranscriptomic library, showing the highest nucleotide identity (82.6%) with cave nectar bat (*Eonycteris spelaea*) gammaretroviruses previously reported in China. The gammaretroviruses detected in both bat species share a 98% nucleotide identity. We constructed a phylogenetic tree based on partial group-specific antigen (gag) genes to determine the evolutionary relationship among retroviruses that we identified here, as well as among other representative gammaretroviruses. Phylogenetic analysis revealed that the *Mops condylurus* and *Hipposideros ruber* gammaretroviruses form a basal evolutionary group consisting of cave nectar bat (*Eonycteris spelaea*) gammaretroviruses, and mongoose (*Galidia elegans*) and short-beaked echidna (*Tachyglossus aculeatus*) reticuloendotheliosis (Figure 5a). The presence of diverse and basal gammaretroviruses in bats suggests that they are a key reservoir species and may have transmitted viruses to other mammals.

*Carboviruses*. In one *Mops condylurus* metatranscriptomic library, we identified three contigs matching different regions of a novel carbovirus RdRp gene, provisionally named *Mops condylurus* carbovirus. In the phylogenetic analysis based on partial RdRp amino acid sequences, this novel carbovirus clustered in a basal position within the *Carbovirus* genus of the family Bornaviridae (Figure 5b). A BLASTx search of the RdRp gene showed that *Mops condylurus* carbovirus exhibited the greatest amino acid similarity (53–57.8%) with the Jungle carpet python virus (*Morelia spilota cheynei*) and the Southwest carpet python (*Morelia spilota imbricata*) virus, the only members of the *Carbovirus* genus isolated previously from Australia [27]. This is the first report of a member of Bornaviridae family described in bats.

*Paramyxoviridae*. Three novel paramyxoviruses were detected in three metatranscriptomic libraries, each from different bat species (*Mops condylurus*, *Hipposideros ruber*, and *Hipposideros jonesi*). We recovered partial protein sequences sharing 76–85% similarity and about 78% similarity to other bat paramyxoviruses from several African countries. Phylogenetic analysis based on partial RdRp amino acid sequences of the novel paramyxoviruses detected in this study and on other previously identified representative members of Paramyxoviridae revealed that they belong to the unclassified Paramyxoviridae group, which forms a distinct and well-defined lineage of bat-related viruses within the Paramyxoviridae phylogeny (Figure 5c). These findings suggest an extension of the geographic and host ranges of the members of this virus family, and that bats may have a global role as potential paramyxoviruses reservoirs.

### 3.3. Filovirus Paleoviral Sequences

**Figure 6:**
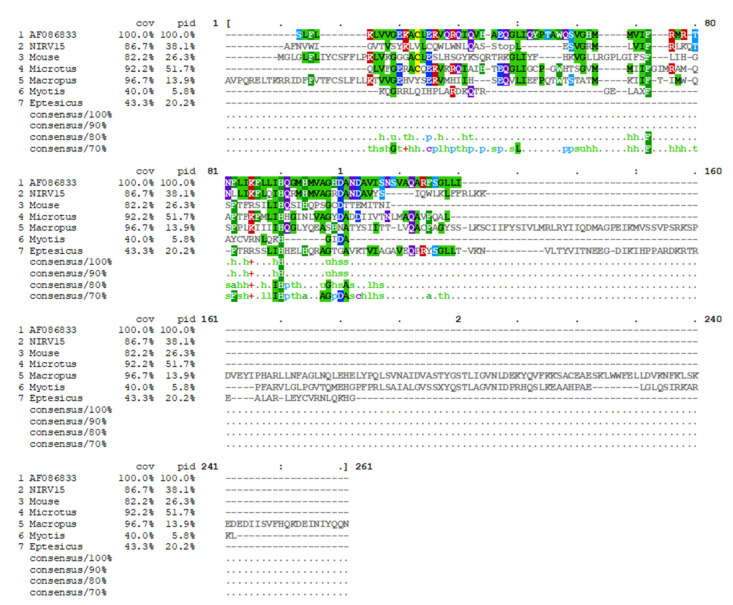
Ebola virus (EBOV)-nonretroviral integrated RNA viruses (NIRVs) alignment and mapping in Guinean bats. Conventional PCR was performed, and the DNA obtained was sequenced and aligned with other EBOV-NIRVs sequences previously described and with EBOV-nucleoprotein (NP) (AF086833).

Considering the high frequency of filovirus-derived NIRVs identified in bats with annotated genomes, we wondered whether other, less well-characterized bat species could also contain NIRVs. To test this hypothesis, we sought to determine the presence of NIRVs in genomic DNA samples obtained from five bat species from which tissue samples were available. To search for NIRVs, we designed “universal” primers directed against NIRV regions that are highly conserved among bats, rodents, and marsupials. Using these primers, we conducted PCR-based screenings of all available samples and obtained an amplicon using template genomic DNA from *Mops condylurus* spleen samples. We then performed conventional Sanger-based sequencing of the amplicon, in silico translation, and identification of ORFs. Multiple protein sequence analysis using Ebola virus nucleoprotein (NP) (Mayinga variant, AF086833) as reference was carried out. Protein alignment analysis revealed the presence of a stop codon in position 46, which indicated that, in contrast to other NIRVs found, for example, in *Myotis* bats, the *Mops* NIRV (NIRV15) does not contain an intact ORF. Strikingly, the 81–107 region of NIRV15 has a higher homology with EBOV NP than any other known NIRVs (Figure 6). Our findings increase the portfolio of known integrated filovirus pseudogenes found in nature, and suggest that NIRVs may be very common in bat genomes.

## 4. Discussion

In March 2014, an outbreak of EVD was detected for the first time in West Africa [28]. Prior evaluation of virus genome sequences suggested that the outbreak probably started in the village of Meliandou around December 2013 [29]. The outbreak was hypothesized as originating as a single spillover event from *Mops condylurus* into humans [30]. Four years after that hypothetical transmission event, we came back to that forest region of the Guéckédou prefecture in Guinea to obtain samples from *Mops* bats as well as other bat species in this area. The goal was to provide a glimpse of viruses circulating in bats in this region of Guinea, and to assess the putative presence of filovirus-derived sequences in bat genomes.

Importantly, some of the sampling sites in this study are houses and construction sites that provide suitable environments for frequent close contact between humans and bats. In fact, one of the most frequent species identified (59 of the 82 bats captured) was *Mops condylurus*. These bats were trapped very frequently in houses and local villages. Although it seems counterintuitive that an abundant bat species may host an elusive virus such as EBOV, *Mops condylurus* were proposed to be responsible not only for the original EBOV spillover that initiated the 2013–2016 epidemic [30], but also for *Bombali ebolavirus*, the latest member of the Ebola virus family that has been recently discovered in *Mops* bats in Sierra Leone and Kenya [9,31,32]. Furthermore, our limited data suggest that *Mops* bats may carry other viruses of the order Mononegavirales, including paramyxoviruses and bornaviruses. These findings suggest that frequent evaluation of the *Mops condylurus* virome may be beneficial for public health welfare.

Knowledge of the species that make up the biodiversity of a territory is crucial in understanding the interplay of its ecosystems, improving their conservation, and being able to better understand the distribution of potential zoonotic viruses. In addition, the analysis of the virome of a given species also provides further insights into viral genetic diversity and the population structure of these viruses in Guinea. Both metagenomics and metatranscriptomics identified a wide diversity of viruses in the bat species samples. We detected sequences related to at least 10 virus families related to vertebrates, including several bat-related novel viruses, such as flaviviruses, pestiviruses, astroviruses, paramyxoviruses, gammaherpesviruses, and bornaviruses. Thus, the combination of metagenomics and metatranscriptomics may be a powerful tool for obtaining comprehensive insights into the taxonomic and functional profile of viromes in the samples. While some of the viruses identified in this study are genetically related to viral families that include important pathogens for vertebrates, their pathogenic potential in bats remains to be determined. Phylogenetic analyses of the newly identified viruses have provided thorough insights into the evolutionary history of bat-related viruses and their association with virus families of other mammals and other host taxa. Our survey suggests that several Guinean bats carry viruses that could potentially spill over to other mammals.

In addition, we described herein the first EBOV-NIRV in *Mops* bats, which provides additional evidence that filovirus pseudogene integration may be common in bats. Previous in silico studies have described filovirus-derived NIRVs in *Myotis* and *Eptesicus* bats, as well as in several rodent and marsupial species [18,33,34]. Interestingly, similar to previously described NIRVs, NIRV15, described herein, is also derived from the viral NP. Moreover, NIRV15 shares a high homology with the N-terminal of NP, which plays an important role in filovirus replication and the formation of nucleocapsid-like structures [35]. The fact that many different species of rodents, marsupials, and bats have NP-derived pseudogenes integrated as single copies is puzzling, and suggests a putative biological function that we are currently investigating.

Metagenomics and metatranscriptomics have become a powerful tool, capable of characterizing the diversity of viral communities in different ecosystems. There is no doubt that zoonotic viruses will continue to emerge from these species. Understanding the ecology of bat-borne viral pathogens could decrease the emergence of zoonotic disease outbreaks, and warrants further investigation.

## Figures and Tables

**Table 1:** Mist netting coordinates and species captured.

Location	Habitat	Lat (N)	Long (W)	Alt	Species
Tongdou cave	Cave	8°30.867	10°21.226	400	Nycteris macrotis **
Nongoa	Rural core	8°30.389	10°19.580	399	Nanonycteris veldkampii **, Micropteropus pusillus **, Myonycteris leptodon **
Mongo forest	Secondary forest	8°36.540	10°15.375	417	Hipposideros ruber **, Hipposideros jonesi **, Doryrhina cyclops **, Rhinolophus sp. *, Myonycteris angolensis smithii **
Bakama Lela cave	Cave	8°33.105	9°55.697	594	Hipposideros ruber **, Hipposideros jonesi **
Tékoulo	House	8°32.550	9°57.232	550	Mops condylurus **, Chaerephon nigeriae **
Koundou	House	8°38.516	10°24.980	426	Rhinolophus fumigatus **
Koundou forest	Secondary forest	8°37.596	10°24.648	411	Hipposideros ruber **, Hipposideros abae **, Pseudoromicia brunnea **
Kimberlite garden	Rural core	8°33.130	10°8.975	428	Hipposideros ruber **, Mops condylurus **

* <95% similarity in molecular identification (indicating that there is not a close species in GenBank); ** >98% similarity in molecular identification. Alt altitude; Lat latitude; Long longitude.

**Table 2:** Organs screened from bats on which necropsies were performed.

Species	Number	Organs
*Nycteris macrotis*	2	spleen, kidney, liver
*Rhinolophus sp.*	1	spleen, kidney, liver, thymus
*Hipposideros jonesi*	2	spleen, kidney, liver, thymus
*Mops condylurus*	6	spleen, kidney, liver, thymus
*Hipposideros ruber*	3	kidney, liver

## Data Availability

Not applicable.
